# Relationship of the bone phenotype of the Klotho mutant mouse model of accelerated aging to changes in skeletal architecture that occur with chronological aging

**DOI:** 10.3389/fendo.2024.1310466

**Published:** 2024-01-30

**Authors:** Lieve Verlinden, Shanshan Li, Vaishali Veldurthy, Geert Carmeliet, Sylvia Christakos

**Affiliations:** ^1^Clinical and Experimental Endocrinology, KU Leuven, Leuven, Belgium; ^2^Department of Microbiology, Biochemistry and Molecular Genetics, New Jersey Medical School, Rutgers, the State University of New Jersey, Newark, NJ, United States

**Keywords:** aging, Klotho, calcium phosphate, TRPV6, skeletal architecture

## Abstract

**Introduction:**

Due to the relatively long life span of rodent models, in order to expediate the identification of novel therapeutics of age related diseases, mouse models of accelerated aging have been developed. In this study we examined skeletal changes in the male and female *Klotho* mutant (*kl/kl*) mice and in male and female chronically aged mice to determine whether the accelerated aging bone phenotype of the *kl/kl* mouse reflects changes in skeletal architecture that occur with chronological aging.

**Methods:**

2, 6 and 20-23 month old C57BL/6 mice were obtained from the National Institute of Aging aged rodent colony and wildtype and *kl/kl* mice were generated as previously described by M. Kuro-o. Microcomputed tomography analysis was performed *ex vivo* to examine trabecular and cortical parameters from the proximal metaphyseal and mid-diaphyseal areas, respectively. Serum calcium and phosphate were analyzed using a colorimetric assay. The expression of duodenal *Trpv6*, which codes for TRPV6, a vitamin D regulated epithelial calcium channel whose expression reflects intestinal calcium absorptive efficiency, was analyzed by quantitative real-time PCR.

**Results and discussion:**

Trabecular bone volume (BV/TV) and trabecular number decreased continuously with age in males and females. In contrast to aging mice, an increase in trabecular bone volume and trabecular number was observed in both male and female *kl/kl* mice. Cortical thickness decreased with advancing age and also decreased in male and female *kl/kl* mice. Serum calcium and phosphate levels were significantly increased in *kl/kl* mice but did not change with age. Aging resulted in a decline in *Trpv6* expression. In the *kl/kl* mice duodenal *Trpv6* was significantly increased. Our findings reflect differences in bone architecture as well as differences in calcium and phosphate homeostasis and expression of *Trpv6* between the *kl/kl* mutant mouse model of accelerated aging and chronological aging. Although the *Klotho* deficient mouse has provided a new understanding of the regulation of mineral homeostasis and bone metabolism, our findings suggest that changes in bone architecture in the *kl/kl* mouse reflect in part systemic disturbances that differ from pathophysiological changes that occur with age including dysregulation of calcium homeostasis that contributes to age related bone loss.

## 1 Introduction

Aging is a complex process that occurs as physiological changes in body functions and changes at the cellular and molecular level contribute to gradual deterioration of function (1). Changes in bone structure and strength are associated with the aging process. Intestinal calcium absorption is dysregulated with age and can result in hyperparathyroidism and significant bone loss (2). Low fractional calcium absorption is associated with increased risk of fracture in the elderly (2). Calcium absorption is primarily regulated by 1,25-dihydroxyvitamin D_3_ [1,25(OH)_2_D_3_], the hormonally active form of vitamin D_3_, which is produced by two sequential hydroxylations of vitamin D_3_ (at C-25 by CYP2R1 in the liver and at C-1 in the kidney by CYP27B1) (3–5). It has been suggested that the age related decrease in intestinal calcium absorption is due in part to resistance to 1,25(OH)_2_D_3_ (2, 6, 7). In addition to vitamin D, klotho, a coreceptor for FGF23 involved in phosphate and calcium homeostasis, is also important for the maintenance of certain physiological functions with age and for the regulation of mineral metabolism (8). A deficiency in *Klotho* is associated with a state of accelerated aging and it has been characterized, in part, by shortened life span, infertility, skin atrophy, osteoporosis and vascular calcification (9). Klotho is expressed highly in the kidney, parathyroid gland and in the choroid plexus (9, 10). Klotho was found to be an obligate coreceptor for fibroblast growth factor (FGF) 23 after reports indicated that *Fgf23*^-/-^ mice showed a similar aging phenotype as the *Klotho* deficient mouse (11–13). Similar phenotypes were reported compared to both the *kl/kl* mouse (which has a hypomorphic mutation for α klotho which was used in this study) as well as the *Klotho*^-/-^ mouse (which lacks the sequence for the klotho protein) (11–13). FGF23 is a bone derived hormone that promotes phosphate diuresis by reducing phosphate reabsorption via suppression of the activity of type II Na dependent phosphate co-transporters in the proximal tubules in a klotho dependent manner (14–16). Klotho decreases transporter activity by promoting NaPi2a proteolytic degradation (17). Elevated 1,25(OH)_2_D_3_ stimulates production of FGF23 and klotho (3, 14, 15). In a negative feedback mechanism secreted FGF23 activates the FGF receptor bound by klotho in renal tubular cells resulting in the suppression of CYP27B1 and increased expression of CYP24A1, an enzyme involved in the catabolism of 1,25(OH)_2_D_3_ (3, 12, 18). In both the *Klotho* deficient mouse and the *Fgf23*^-/-^ mouse increased levels of 1,25(OH)_2_D_3_ and phosphate and reduced levels of PTH have been reported (13, 19). Thus 1,25(OH)_2_D_3_, PTH and FGF23/klotho act together to regulate calcium and phosphate homeostasis. Understanding the regulation of mineral metabolism and its dysregulation with aging is important to provide insight into mechanisms involved in skeletal aging and to define causes of age related skeletal disease.

Due to the relatively long life span of rodent models, in order to expediate the identification of novel therapeutics of age related diseases, mouse models of accelerated aging have been developed. Since a bone phenotype has been reported in the *Klotho* deficient mouse models of accelerated aging (19–22), in this study we examined skeletal changes in male and female *Klotho* mutant *kl/kl* mice and in male and female chronically aged mice (whose changes in skeletal structure have been reported to be similar to human aging) (23, 24) in order to understand mechanisms involved in bone metabolism and to determine the relationship of the bone phenotype of the *Klotho* deficient mouse to changes in skeletal architecture that occur with age.

## 2 Materials and methods

### 2.1 Animals

C57BL/6 mice (2, 6 and 20 - 23 months old) were obtained from the National Institutes of Aging (NIA) aged rodent colony. *kl/kl* mice were generated by M. Kuro-o by backcrossing the original *kl/+* mice (9) (a hybrid of C3H and B6) with 129S1svlmJ mice for more than 12 generations. Thus the WT (+/+) mice are 129S1SvlmJ and the *kl/kl* mice are on the 129 genetic background and compared to WT (+/+) controls. The mice were analyzed at 6 – 7.5 weeks of age (*kl/kl* mice die prematurely at 8 -9 weeks of age). Mice were maintained in a virus and parasite-free barrier facility, given standard rodent chow diet (Rodent Laboratory Chow 5001; Ralston Purina co., St. Louis, Mo) and water *ad libitum* and exposed to a 12h-light, 12h-dark cycle. Both male and female mice were used. All the animal procedures were approved by the Institutional Animal Care and Use Committee (IACUC) at Rutgers, New Jersey Medical School. Sample sizes of the different experimental groups are indicated in **Supplementary Tables 1**, **2**.

### 2.2 Tissue harvest and serum analysis

Mouse duodenum was rinsed in ice-cold phosphate buffered saline, flash frozen in liquid nitrogen and stored at -80°C. Blood was collected and serum was prepared for analysis of calcium and phosphate using a colorimetric assay (Pointe Scientific, Inc., Canton MI) determined by Heartland Laboratories, Ames, IA. Tibiae were fixed in 2% paraformaldehyde for 24h. Micro-computed tomography (µCT) analysis of the left tibiae was performed as described below.

### 2.3 Bone analysis

µCT analysis was performed *ex vivo* using a high-resolution SkyScan 1172 (50 kV, 200 µA, 0.5-mm aluminum filter, 0.6° rotation step, 5 µm pixel size) to examine trabecular and cortical bone parameters (25). Serial tomographs, reconstructed from raw data using the cone-beam reconstruction software (NRecon,v.1.4.4.0; Skyscan with following settings: smoothing:0; ring artifact reduction: 7; beam hardening: 30%) with global thresholding. The thresholds set to detect trabecular bone were 80-255 and for cortical bone 90-255. Volumes of interest for 3D morphometric analysis were between 0.85 and 2.35 mm distal to the growth plate for trabecular analysis and between 3 and 3.5 mm distal to the growth plate for cortical analysis. Analysis was performed according to the guidelines of the American Society for Bone and Mineral Research (26).

### 2.4 RNA isolation and expression analysis

Total RNA was isolated from mouse duodenum using Ribozol RNA extraction reagent (Amresco, Solon, OH) or TRizol reagent (Invitrogen, Carlsbad, CA) according to the manufacturer’s instructions and subsequently purified with an RNeasy Plus universal kit (Qiagen, Hilden, Germany) using on-column DNase digestion (Qiagen). RNA concentration was measured with a NanoDrop spectrophotometer (ND-1000; Isogen, Life Science, Utrecht, The Netherlands), RNA integrity was assessed using a denaturing agarose gel stained with ethidium bromide or by a bioanalyzer nanochip (Agilent Technologies, Santa Clara, CA). For quantitative real-time PCR (qRT-PCR), 2 µg of total RNA was used to synthesize cDNA using a Superscript III first-strand synthesis system (Invitrogen) according to the manufacturer’s instructions. Relative quantification of target gene expression was performed using TaqMan analyses. Mm00499069-m1 TaqMan gene expression probe (Applied Biosystems, Foster City, CA) was used for qRT-PCR analysis of *Trpv6.* The cycle steps were as follows: an initial 2-min incubation at 50°C, and 10 min at 95°C followed by 40 cycles of 95°C for 15 s; 60°C for 60 s. Expression levels of *Trpv6* were normalized to *Gapdh* (Mm999999-g1). The comparative threshold cycle (2^-ΔΔCT^) method was used to calculate relative gene expression.

### 2.5 Statistical analysis

Results are displayed in the figures as means ± standard deviations of the means (SD). Additional information on the experimental groups [sample size, 95% confidence intervals (CI), effect sizes are summarized in **Supplementary Table 1** for the aging mice and **Supplementary Table 2** for the *kl/kl* mice and their wildtype littermates]. To consider significant difference between groups, data were analyzed using Student’s t test, with Welch’s correction in case of unequal variances, or analysis of variance (ANOVA) followed by Tukey’s multiple comparisons tests.

## 3 Results

### 3.1 Relationship of the bone phenotype of the *kl/kl* mouse to changes in skeletal architecture that occur with chronological aging

**Figure 1:**
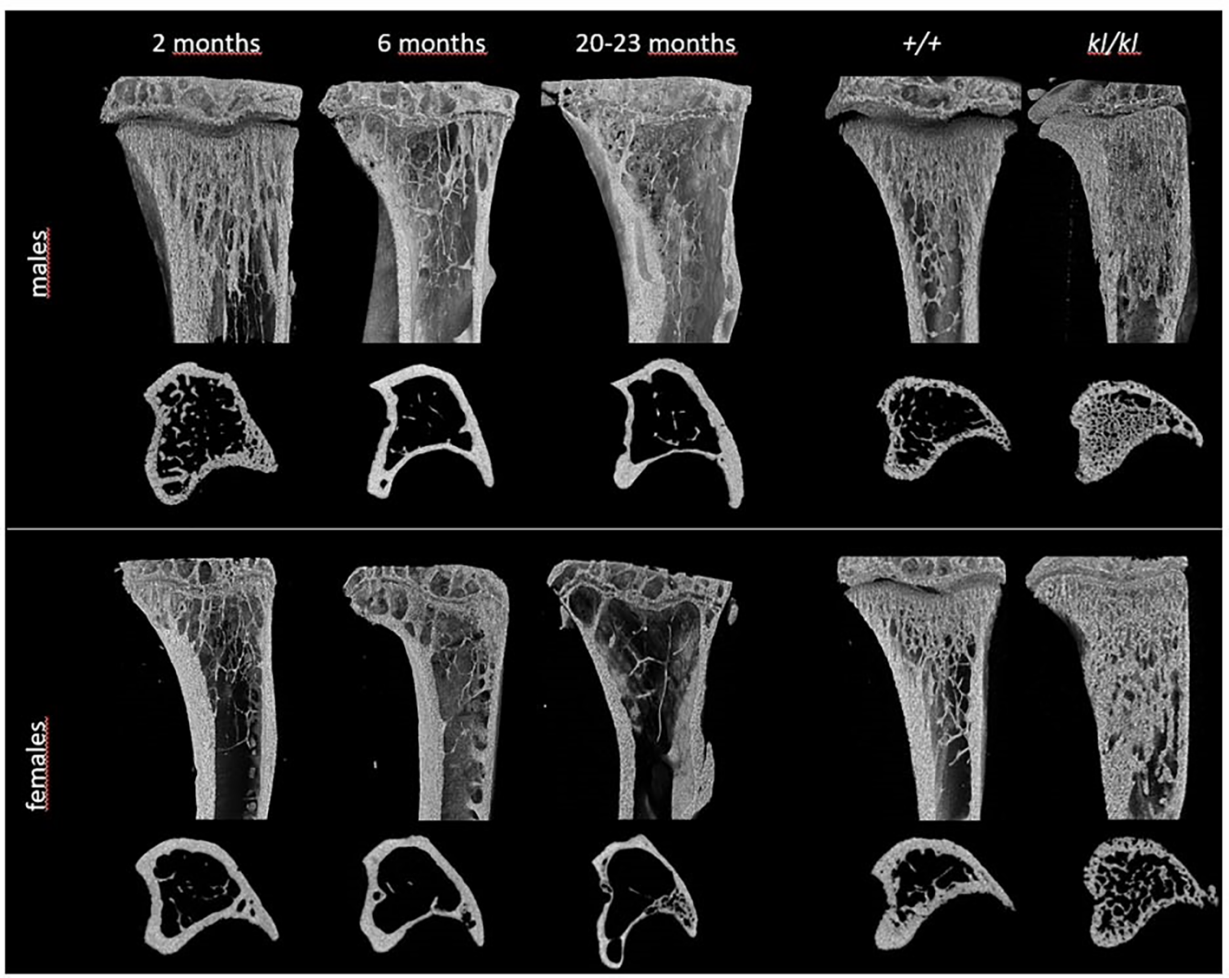
Representative 3D models of tibial epiphyses and metaphyses and cross-sections of the tibial mid-diaphysis from aging (left panels) and *klotho* wildtype (*+/+*) and deficient (*kl/kl*) mice (right panels).

**Figure 2:**
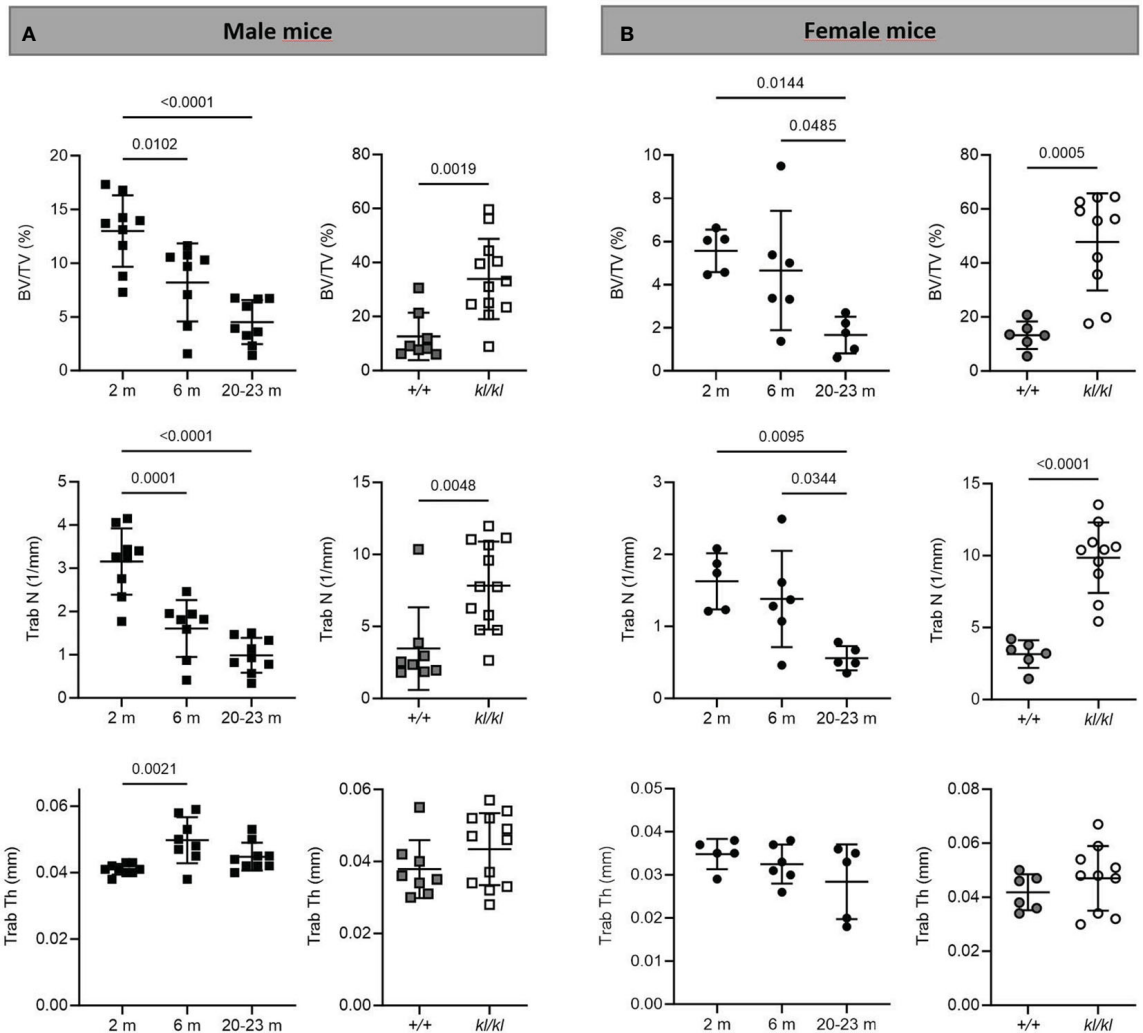
Trabecular analysis in aging and in *klotho* wildtype and deficient mice. Trabecular bone volume (BV/TV), trabecular number (Trab N), and trabecular thickness (Trab Th) were determined by µCT analysis in tibia of male **(A)** and female **(B)** aging and *klotho* wildtype (*+/+*) and deficient (*kl/kl*) mice (n = 5-12). Data are expressed as mean and SD. One-way ANOVA analysis, followed by Tukey’s multiple comparisons test, was applied to detect significant effects between the different age groups and student’s t-tests were performed to identify significant differences between *klotho* wildtype (*+/+*) and deficient (*kl/kl*) mice.

**Figure 3:**
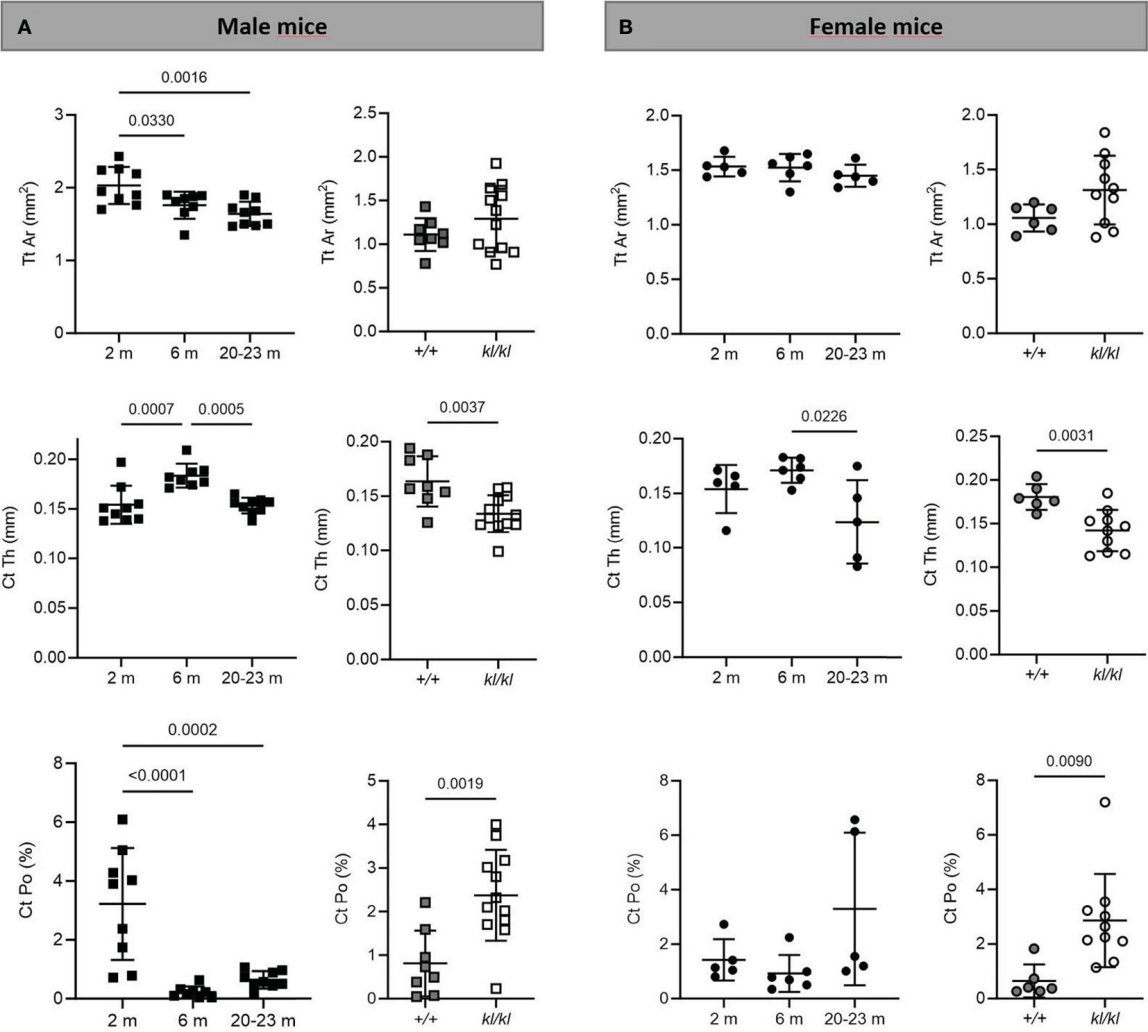
Cortical analysis in aging and in *klotho* wildtype and deficient mice. Mean total cross-sectional tissue area (Tt Ar), cortical thickness (Ct Th), and cortical porosity (Ct Po) were determined by µCT analysis in tibia of male **(A)** and female **(B)** aging and *klotho* wildtype (*+/+*) and deficient (*kl/kl*) mice (n = 5-12). Data are expressed as mean and SD. One-way ANOVA analysis, followed by Tukey’s multiple comparisons test, was applied to detect significant effects between the different age groups and student’s t-tests were performed to identify significant differences between *klotho* wildtype (*+/+*) and deficient (*kl/kl*) mice.

To investigate whether the accelerated aging bone phenotype of the *Klotho* deficient mouse reflects changes in skeletal architecture that occur with chronological aging, changes in bone architecture in the tibia with age (2 months, 6 months and 20-23 months) and in wildtype (*+/+*) and *Klotho* mutant (*kl/kl*) mice were assessed by µCT analysis. Cross-sectional 3D-analysis of the tibia indicated a progressive decline in bone mass with chronological aging in both males and females and indicated cortical thinning with age, whereas increased cortical porosity was observed in the *kl/kl* mice in both males and females compared to wildtype (*+/+*) controls (**Figures 1**). Trabecular BV/TV decreased continuously with age in both males and females (**Figures 2A, B**, upper left panels). BV/TV in males decreased 37% between 2 and 6 months and 65% between 2 and 20-23 months. The change in BV/TV between 6 and 20- 23 months was not significant in males (**Figure 2A**, upper left panel). In females significant decreases in BV/TV were observed between 2 and 20-23 months (70%) and between 6 and 20-23 months of age (65%) (**Figure 2B**, upper left panel). Changes with chronological age in trabecular number followed a similar pattern as BV/TV in both males and females (**Figures 2A, B** middle left panels). Significantly lower BV/TV and trabecular number was observed in females compared to males at 2 months of age (**Supplementary Figure 1**). In contrast to chronological aging, trabecular volume and trabecular number were increased in both male and female *kl/kl* mice compared to wildtype (+/+) mice [**Figures 2A, B** upper right panel: BV/TV (169% and 268% increase in males and females, respectively) and **Figures 2A, B**, middle right panel: Trab N (135% and 215% increase in males and females, respectively)]. No significant differences between males and females were detected for wildtype (*+/+*) and *Klotho* deficient (*kl/kl*) mice (**Supplementary Figure 1**). Trabecular thickness increased in males between 2 and 6 months of age but with increasing age remained constant in both males and females (**Figures 2A, B** lower left panels). There were no significant changes in trabecular thickness in male or female *kl/kl* mice compared to wildtype mice (*+/+*) (**Figures 2A, B**, lower right panels). With regard to cortical bone, there were no changes in total cross-sectional tissue area with advancing age in males and females and in male and female *kl/kl* mice compared to +/+ mice (**Figures 3A, B** upper panel). Cortical thickness increased significantly between 2 and 6 months in males and then decreased with advancing age in both males and females (**Figures 3A, B** middle left panels). A decrease in the thickness of cortical bone was also observed in both male and female *kl/kl* mice (**Figures 3A, B** middle right panels). Cortical porosity decreased with age in males, was unchanged in females and was significantly increased in male and female *Klotho* deficient (*kl/kl*) mice (192% and 340% respectively, **Figures 3A, B**, lower panels).

### 3.2 Serum data and duodenal *Trpv6* expression

**Figure 4:**
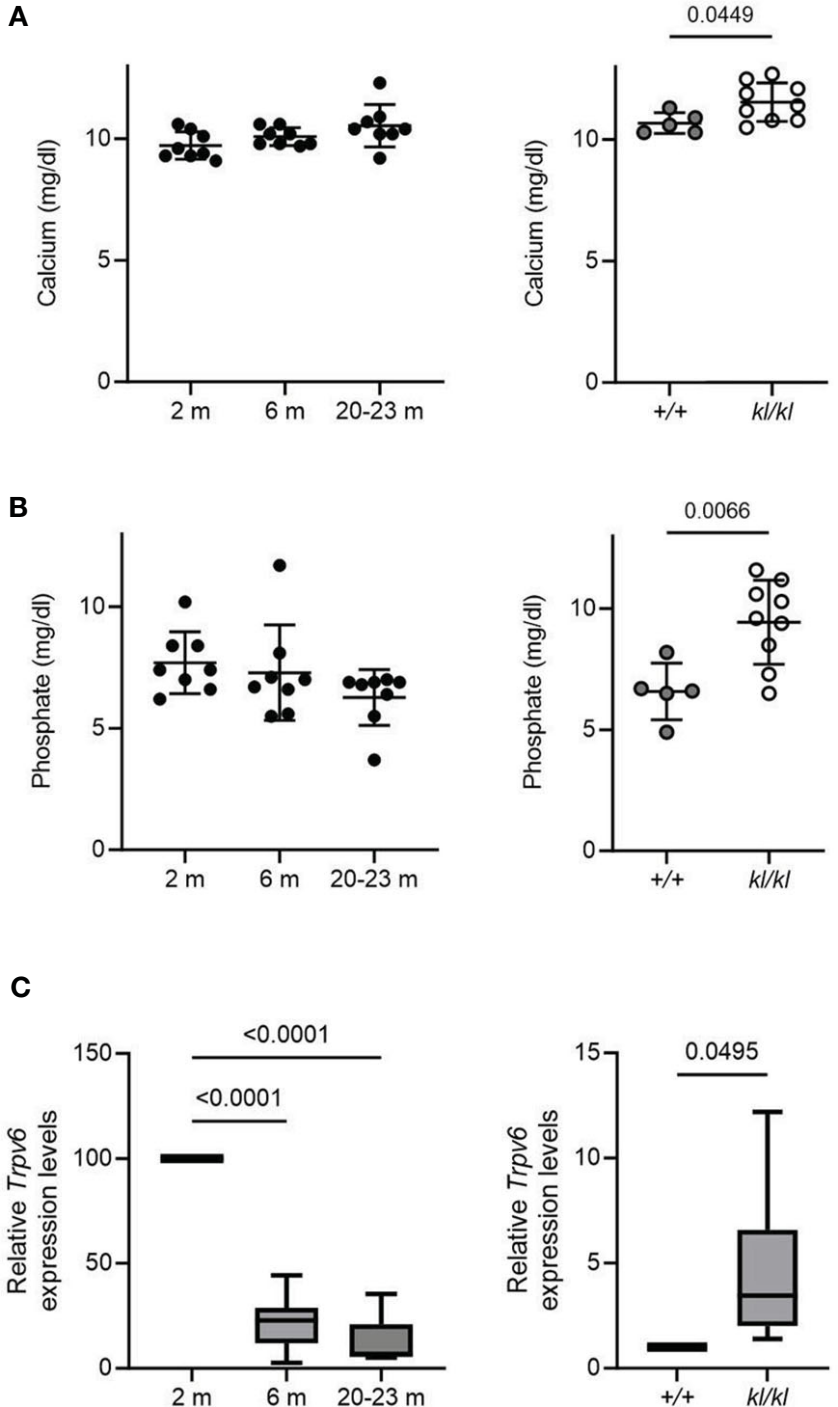
Serum calcium and phosphate and duodenal *Trpv6* expression in aging and in *klotho* wildtype and deficient mice. Serum calcium **(A)** and phosphate **(B)** concentrations were determined in aging and in *klotho* wildtype (*+/+*) and deficient (*kl/kl*) mice. Data from male and female mice were pooled (n = 5-9). **(C)** *Trpv6* transcript levels in duodenum were determined by qPCR analysis in aging and in *klotho* wildtype (*+/+*) and deficient (*kl/kl*) mice. Data from male and female mice were pooled (n = 6-8). All data are expressed as mean and SD. One-way ANOVA analysis, followed by Tukey’s multiple comparisons test, or student’s t-tests were performed to identify significant differences.

There were no significant differences with age in serum calcium and phosphate levels (**Figures 4A, B**, left panels). Serum calcium and phosphate levels were significantly increased in the *kl/kl* mice (**Figures 4A, B**, right panels). Low bone density with age has been associated with intestinal calcium malabsorption, which has been suggested to be due in part to resistance to 1,25(OH)_2_D_3_ (2, 6, 7). Therefore, we examined the expression of duodenal *Trpv6*, which codes for TRPV6 an epithelial calcium channel whose expression reflects calcium absorptive efficiency and is considered a rate limiting step in the process of vitamin D dependent intestinal calcium absorption (4, 27, 28). Aging resulted in a decline in *Trpv6* expression (**Figure 4C**, left panel). However, in *Klotho* deficient (*kl/kl*) mice duodenal *Trpv6* was significantly increased compared to wildtype mice (*+/+*) (**Figure 4C**, right panel).

## 4 Discussion

The *kl/kl* mouse discovered by Kuro-o in 1997 (9) was the first animal model caused by a single mutation that displayed many features of age associated disease. A major contribution to the discovery of α klotho and its function as a coreceptor for FGF23 is that it resulted in new insights that have changed the concepts related to mechanisms involved in the regulation of mineral homeostasis. Elevations in FGF23 in order to maintain phosphate excretion and a decline in klotho, which can cause a compensatory increase in FGF23, have been reported to be early events in CKD (29–31). These findings have led to a new understanding of mechanisms involved in the pathophysiology of CKD. In addition, the potential for klotho together with other known treatments to attenuate age associated pathologies and as a biomarker for certain diseases including renal, cardiovascular and neurodegenerative diseases has been suggested (32, 33). In this study in order to understand mechanisms involved in skeletal aging we examined the bone architecture of the *Klotho* deficient mouse and its relationship to changes in skeletal architecture that occur with chronological age. Our findings reflect differences in bone architecture as well as differences in calcium and phosphate homeostasis and expression of *Trpv6* involved in intestinal calcium absorption between the *Klotho* deficient (*kl/kl*) model of accelerated aging and the chronologically aged mouse. Although the *kl/kl* mouse has provided a new understanding of the regulation of mineral homeostasis and bone metabolism and a model of premature aging that includes atherosclerosis and infertility, our findings suggest that changes in bone architecture in the *kl/kl* mouse reflect in part systemic disturbances that differ from pathophysiological changes that occur with age including dysregulation of calcium homeostasis that contribute to age related bone loss.

Our results showed marked changes in bone architecture with age as well as in the *kl/kl* mouse. Micro CT analysis of tibia in aging mice showed that trabecular bone volume and trabecular number decreased with age in both sexes. A significant decrease in females compared to males in BV/TV and trabecular thickness was observed at 2 months. Cortical thickness decreased with advancing age in both sexes. Trabecular thickness remained relatively constant in mice with age which may be due to a compensatory mechanism. In human aging in both sexes, similar to our studies in mice, elderly individuals have been reported to have lower BV/TV accompanied by a reduction in cortical thickness and in most studies in a reduction in trabecular number (34–36). However, unlike findings observed in the elderly. an age-related reduction in trabecular thickness was not observed in mice. The changes we observed in bone architecture in aging mice as well as relatively constant trabecular thickness are similar to what has been reported by Halloran and Glatt (23, 24). Although collectively our data are similar to changes in skeletal architecture observed in the elderly, differences observed may be due in part to different mechanisms of bone loss. In the *kl/kl* mouse, although decreased cortical thickness and increased cortical porosity were observed, trabecular volume and number were increased which is in contrast to the pattern of bone impairment in mouse and human aging. Intestinal *Trpv6* declined with age which is consistent with the decrease in *Trpv6* reported in the 12 month old mouse and the decline in intestinal calcium transport as a function of age previously reported (37, 38). The decline in *Trpv6* with age suggests that the decrease in *Trpv6* may be one factor involved in intestinal malabsorption with age that contributes to age related bone loss. However intestinal *Trpv6* was increased in the *kl/kl* mouse. The increase in *Trpv6* may be due in part to the increase in 1,25(OH)_2_D_3_ levels that have been observed in the *kl/kl* mouse (19). An increase in *Trpv6* may reflect metabolic dysfunction in the *kl/kl* mice arising in part from excess intestinal calcium absorption compared to chronological aging and reflected by an increase in serum calcium (**Figure 4**).

In order to understand the mechanisms that result in the bone defects observed in the *kl/kl* mouse, Yamashita et al. (39) used a bone marrow ablation model. They noted a site-specific reduction in the number and size of osteoclasts as well as high expression of osteoprotegerin (OPG), an inhibitor of osteoclastogenesis and osteoclast function. They concluded that the abnormal trabecular bone structure is due to part to a defect in bone resorption and that the *kl/kl* mice exhibit an osteopetrotic as well as an osteopenic phenotype. An earlier study by Kawaguchi et al. suggested that the phenotype of the *kl/kl* mouse is due to independent impairment of osteoblast as well as osteoclast differentiation (40).

Different bone phenotypes have been reported for the *Klotho* deficient mice. Kaludjerovic and Lanske reviewed the findings from several research groups that have independently investigated the bone phenotype of *Klotho* deficient mice (20). They concluded that although it had been reported that *Klotho* deficient mice have osteoporotic bones, the common observation across these studies was that the *kl/kl* mouse as well as the *Klotho*^-/-^ mouse have high trabecular bone volume, similar to our findings. The authors suggested that the different bone phenotypes reported for the *Klotho* deficient mice may be due in part to analysis of different bone regions and site-specific changes in the bones of the *Klotho* deficient mice. Mature osteocyte specific knock down of *Klotho* was shown to result in significantly higher trabecular volume and connectivity in 5-week-old animals compared to healthy controls (41, 42). This finding indicates a role for klotho in the bone independent of the endocrine effects on bone due to global *Klotho* deficiency and that klotho is a negative regulator of bone formation.

With regard to further mechanisms involved in the *kl/kl* accelerated aging model, it was noted that the aging symptoms of the *kl/kl* mice were alleviated when the mice were fed a vitamin D-deficient or low phosphate diet indicating an underlying metabolic dysfunction arising from excess phosphate or 1,25(OH)_2_D_3_ (43, 44). Whether excess phosphate or 1,25(OH)_2_D_3_ is responsible for the aging phenotype had been a matter of debate (44, 45). Studies showing that ablation of the Napi2a gene from *Klotho*^-/-^ mice result in reduction or elimination of soft tissue calcification even in the presence of high 1,25(OH)_2_D_3_ and calcium levels suggested that retention of phosphate may be one key factor involved in accelerated aging in the *Klotho^-/-^* deficient mouse model (46). It was also suggested that the metabolic dysfunction in *kl/kl* mice may be due to both increased calcium and phosphate resulting in calcium phosphate precipitates and calciprotein particles that can induce cell damage and inflammation (8).

Since the discovery of the *Klotho* deficient models additional murine models of accelerated aging have been developed which may provide new insight into mechanisms involved in age related diseases including age related skeletal disease (47–50). One model that has been found to reflect natural aging is the *Ercc1*^-/Δ^ model which carries mutations in the ERCC-XPF exonuclease, important for multiple DNA repair pathways (48). The *Ercc1*^-/Δ^ mice develop many age-related diseases including severe and progressive osteoporosis, premature senescence of osteoblastic progenitors and enhanced osteoclastogenesis (48). At 22 weeks of age the mice had > 60% reduction in BV/TV, reduced trabecular thickness and an increase in trabecular space compared to WT controls demonstrating the importance of ERCC1-XPF dependent DNA repair for maintaining normal bone homeostasis (48). Another marker of aging is telomerase dysfunction, a cause of cellular senescence. The accelerated aging mouse model of telomere dysfunction (*Terc*^-/-^ mice; deletion of telomerase reverse transcriptase) was suggested as a model for human bone aging since at three months of age *Terc^-^*^/-^ mice had significant decreases in BV/TV, trabecular number, trabecular thickness, increased trabecular spacing as well as decreased cortical thickness and increased porosity. These skeletal changes became more pronounced with age. Osteoblast dysfunction was noted as the primary mechanism for osteoporosis in these mice (47). Although further studies are needed these findings suggest that mice with defects in telomerase maintenance may be an additional useful model for studying age related osteoporosis.

In summary, studying mouse models of accelerated aging has provided new insight into mechanisms involved in multiple pathologies including age related skeletal disease. The *Klotho* deficient mouse model provided a new understanding of the regulation of mineral homeostasis and bone metabolism. There is a strong rationale for the use of additional mouse models of accelerated aging which mimic changes observed with human aging. It should be noted that each mouse model may reflect different traits related to skeletal changes that occur with human aging. Future studies are needed to determine which factors, identified using models of accelerated aging, can be potential targets for therapeutic approaches to delay skeletal aging.

## Data availability statement

The original contributions presented in the study are included in the article/**Supplementary Material**. Further inquiries can be directed to the corresponding author.

## Ethics statement

The animal study was approved by Institutional Animal Care and Use committee Rutgers, New Jersey Medical School. The study was conducted in accordance with the local legislation and institutional requirements.

## Author contributions

LV: Conceptualization, Data curation, Formal Analysis, Investigation, Writing – original draft, Writing – review & editing. SL: Conceptualization, Data curation, Formal Analysis, Investigation, Writing – original draft, Writing – review & editing. VV: Conceptualization, Data curation, Formal Analysis, Investigation, Writing – original draft, Writing – review & editing. GC: Conceptualization, Funding acquisition, Supervision, Writing – review & editing. SC: Conceptualization, Data curation, Formal Analysis, Funding acquisition, Investigation, Supervision, Writing – original draft, Writing – review & editing.

## Supplementary material

Supplementary Figure 1 Comparison of trabecular bone volume and number between male and female mice. Direct comparison of tibial µCT analysis of trabecular bone volume **(A)** and trabecular number **(B)** between male (black bars) and female (grey bars) aging and *klotho* wildtype (*+/+*) and deficient (*kl/kl*) mice (n = 5-12). Data are expressed as mean and SD. Two-way ANOVA analysis followed by Tukey’s multiple comparisons test. ^#^*p* < 0.05 vs. 2 and 6 months; ^+^*p* < 0.05 vs. 2 months; **p* < 0.05 compared to +/+ (WT) mice in that same sex, $ vs male mice of the same age.
