# Supplementary figures and images for: Relationship of the bone phenotype of the Klotho mutant mouse model of accelerated aging to changes in skeletal architecture that occur with chronological aging
Source: Front Endocrinol (Lausanne). 2024 Jan 30;15:1310466. doi: 10.3389/fendo.2024.1310466 (PMC10861770; doi:10.3389/fendo.2024.1310466)

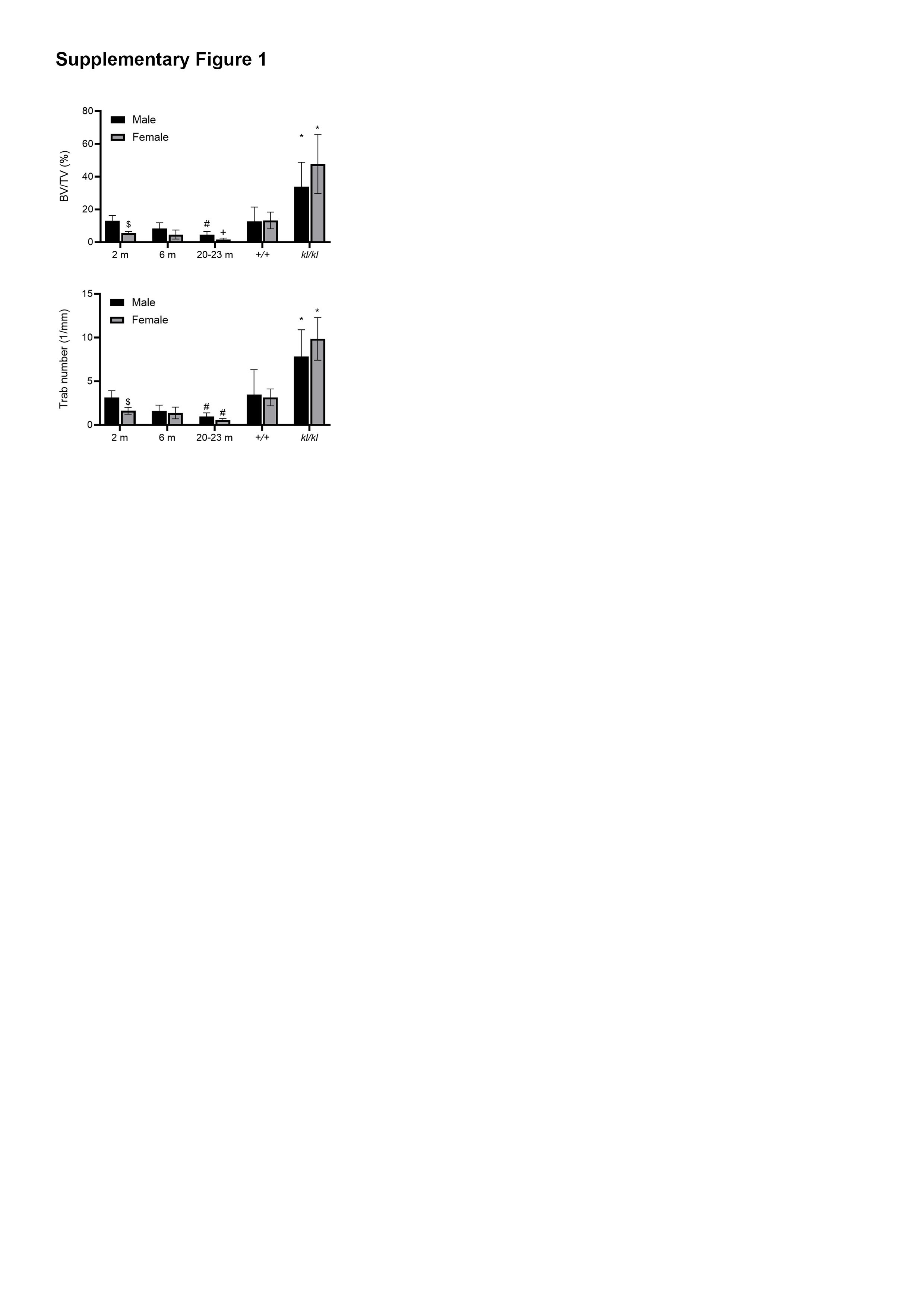

Supplement: Supplementary Figure 1 — Comparison of trabecular bone volume and number between male and female mice. Direct comparison of tibial µCT analysis of trabecular bone volume (A) and trabecular number (B) between male (black bars) and female (grey bars) aging and klotho wildtype (+/+) and deficient (kl/kl) mice (n = 5-12). Data are expressed as mean and SD. Two-way ANOVA analysis followed by Tukey’s multiple comparisons test. #p < 0.05 vs. 2 and 6 months; +p < 0.05 vs. 2 months; *p < 0.05 compared to +/+ (WT) mice in that same sex, $ vs male mice of the same age. [file Image_1.jpeg]
