# Supplementary material for: Relationship of the bone phenotype of the Klotho mutant mouse model of accelerated aging to changes in skeletal architecture that occur with chronological aging
Source: Front Endocrinol (Lausanne). 2024 Jan 30;15:1310466. doi: 10.3389/fendo.2024.1310466 (PMC10861770; doi:10.3389/fendo.2024.1310466)
Supplement: Supplementary file 3 [file Table_2.docx]

Supplemental table 2. Summary of µCT and serum biochemical data of *kl/kl* mice
